# Tumor Microenvironment Triggered In Situ Coagulation of Supramolecularly Engineered Platelets for Precise Tumor Embolization

**DOI:** 10.1002/advs.202414879

**Published:** 2025-04-07

**Authors:** Junyan Li, Ziyi Wang, Ruifeng Luo, Xingping Quan, Hong U Fong, Qian Cheng, Jianwen Wei, Leo Wang, Yonghua Zhao, Ruibing Wang

**Affiliations:** ^1^ State Key Laboratory of Quality Research in Chinese Medicine Institute of Chinese Medical Sciences University of Macau Taipa Macao SAR 999078 China; ^2^ Kitsilano Secondary School Vancouver BC V6K 2J6 Canada; ^3^ MoE Frontiers Science Centre for Precision Oncology University of Macau Taipa Macao SAR 999078 China

**Keywords:** cell carriers, embolization therapy, morphology transformation, platelets, supramolecular

## Abstract

Although embolization therapy has demonstrated success in impeding tumor growth, concerns persist regarding potential tumor recurrence and inadvertent embolization of non‐target tissues. In this study, drawing inspiration from the natural targeting and coagulation process of platelets in injured blood vessels, platelets are engineered by integrating acid‐sensitive, morphology‐transformable nanoparticles onto their surface through supramolecular conjugation (PLT‐NP). The nanoparticles are constructed through the self‐assembly of a β‐amyloid derived peptide (FFVLK) terminally functionalized with Fmoc, hexahistidine (His_6_), and a polyethylene glycol (PEG)‐functionalized cyclodextrin (CD). The supramolecularly engineered platelets actively accumulate in the tumor tissue upon inducing a tumor blood vessel injury through tumor resection. In response to the local acidic microenvironment, the nanoparticles undergo a morphological transformation into nanofibers via spontaneous assembly of FFLVK into fibril structures through hydrogen bonding and β‐sheet interactions, to artificially enhance the coagulation and aggregation of platelets, causing occlusion of tumor blood vessels. The supramolecularly engineered platelets efficiently embolize tumor blood vessels in a specific manner, effectively suppressing tumor growth, metastasis, and recurrence, thus offering a promising paradigm for combating cancer.

## Graphical abstract

Occlusion of tumor blood vessels represents a promising yet challenging approach in cancer treatment. Herein, supramolecularly engineered platelets conjugated with morphology‐transformable nanoparticles is developed for precise tumor embolization. The engineered platelets actively accumulate at tumor sites following induced blood vessel injury. In response to the acidic tumor microenvironment, the nanoparticles conjugated with platelets transformed into nanofibers, crosslinking platelets and promoting the artificial coagulation and aggregation of platelets, leading to the specific occlusion of tumor blood vessels.

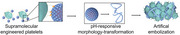

## 1 Introduction

Selective occlusion of tumor blood vessels, depriving tumors of oxygen and nutrients, has demonstrated potential in inducing tumor necrosis and shrinkage,^[^ ^1^ ^]^ which is an appealing strategy against cancer and offers advantages over conventional treatments, such as minimal invasiveness, broad applicability, and coagulation cascades,^[^ ^2^ ^]^ augmenting its significance in cancer management. Various embolization techniques have been developed in clinical applications, for instance, interventional catheter‐based embolization is a mainstream clinical approach for treating specific hypervascular cancers.^[^ ^3^ ^]^ However, the associated complications (e.g., embolization syndromes and accidental damage to healthy tissues) as well as requirements of catheter placement still limited the application of this technique in only very few types of tumors, e.g. liver tumor. It is therefore imperative to pursue new‐generation strategies to occlude tumor blood vessels precisely and adequately for efficient and safe cancer treatment without the need of catheter placement. Vascular coagulation agents conjugated with targeting ligands have been applied to induce thrombosis and tumor necrosis.^[^ ^4^ ^]^ More recently, researchers have drawn inspiration from the natural coagulation process orchestrated by platelet activation and fibrin formation. Platelet‐mimicking or morphology‐transformable nanoformulations were employed to create fibrous networks within the tumor microenvironment. This innovative approach disrupts the supply of nutrients to tumors and effectively halts their growth.^[^ ^5^ ^]^ For instance, Wang et al developed biomimetic fibrin using self‐assembling peptides, which actively target tumor neovascular endothelial cells, initiating artificial coagulation within tumor regions.^[^ ^6^ ^]^ In a separate study, Zhao and Nie et al. designed a programmable DNA nanorobot for the targeted transport of thrombin, inducing infarction in tumor‐associated vessels.^[^ ^7^ ^]^ These pioneering endeavors have sparked subsequent investigations centered on stimuli‐responsive morphology‐transformable nanoparticles.^[^ ^8^ ^]^ However, it's worth noting that nanoparticles administered intravascularly face intricate biological clearance and various barriers before reaching their intended tumor sites,^[^ ^9^ ^]^ resulting in unsatisfactory accumulation in tumor tissues, even when modified with active targeting ligands.^[^ ^10^ ^]^ Furthermore, the inherent immunogenicity of nanodrugs poses challenges in embolization therapy, as nonselective thrombosis may lead to coagulation events and severe side effects in healthy tissues. Therefore, the quest for suitable carriers that can enable precise delivery of embolic materials and the controlled induction of thrombosis within tumor tissues is of utmost importance.

On the other hand, cell‐based therapies offer several inherent advantages, including exceptional biocompatibility, prolonged circulation, and being their “self” nature, allowing them to efficiently evade RES clearance.^[^ ^11^ ^]^ Cell‐based systems, including hemocytes, immune cells, and genetically engineered stem cells have been harnessed to augment the pharmacokinetic and pharmacodynamics properties of drugs.^[^ ^12^ ^]^ Platelets, as the second most prevalent blood cells, play pivotal roles in hemostasis, thrombosis, and disease progression, particularly in the context of tumor progression and metastasis.^[^ ^13^ ^]^ In cancer therapy, several platelet‐based therapies are currently undergoing various stages of preclinical or clinical trials. For instance, Gu et.al utilized platelet‐conjugated anti‐PDL1 to leverage the wound‐targeting properties of platelets, promoting the accumulation of anti‐PDL1 at the surgical site of tumor. This approach has shown promise in reducing post‐surgical tumor recurrence and metastasis.^[^ ^14^ ^]^ Beyond their physiological role in surgical wound targeting, platelets also play crucial roles in the thrombosis and hemostatic processes by rapidly forming blood coagulation at the site of vessel injury.^[^ ^15^ ^]^ Following the formation of the platelet plug, the coagulation cascade is initiated, resulting in the formation of a stable fibrous clot.^[^ ^16^ ^]^ Given their natural and superior coagulation ability, it is an ideal approach to harness platelet‐based strategies for cancer embolization therapy.

**Scheme 1:**
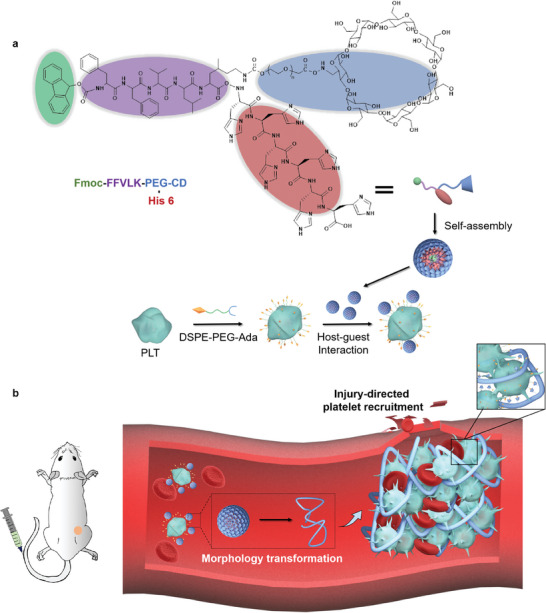
The scheme of supramolecular platelet‐nanoparticles conjugates (PLT‐NP) for tumor‐targeting and in situ coagulation to embolize tumor vessels. a) Molecular structures of the hydrogen‐bonding peptide‐based system consisting of four motifs, which self‐assembles into nanoparticles in an aqueous solution (pH = 7.4). The self‐assembled nanoparticles further bond with Ada‐factionalized platelet via host‐guest interactions. b) PLT‐NP actively targets the tumor vessels via wound‐directed platelet recruitment. In the acidic microenvironment of tumor, the nanoparticles could transform into a fibrous network, crosslinking platelets and trapping RBCs within tumor vessels in situ, for selective and effective tumor suppression.

In this study, inspired by the natural coagulation process and the physiological functions of platelets, which play a crucial role in injury‐directed recruitment and cascade clotting, we developed supramolecularly engineered platelets (supramolecular platelet‐nanodrug conjugate) to precisely and effectively block tumor blood vessels (**Scheme** **1**). In this design, we utilize nanoparticles constructed through the self‐assembly of a functionalized peptide (FFVLK). This system incorporates four distinct motifs (Figure S1, Supporting Information): i) A hydrophobic 9‐fluorenylmethyloxycarbonyl (Fmoc) motif; ii) A peptide segment known as Phe‐Phe‐Val‐Leu‐Lys (FFVLK), which is the reverse sequence of KLVFF. This FFVLK segment is derived from β‐amyloid (Aβ) and is well‐known for its propensity to adopt a β‐sheet conformation, leading to spontaneous assembly into fibril structures through hydrogen bonding and β‐sheet interactions;^[^ ^17^ ^]^ iii) Hexahistidine (His_6_), a peptide sequence that undergoes a hydrophobic‐to‐hydrophilic transition in response to acidic conditions, typically occurring ≈pH 6.5;^[^ ^5b^ ^]^ iv) A hydrophilic polyethylene glycol (PEG) chain functionalized with the macrocyclic molecule cyclodextrin (CD). This amphiphilic peptide‐based system, referred to as Fmoc‐FFVLK‐His_6_‐PEG‐CD (FHPC), naturally assembles into nanoparticles (NPs) when introduced into an aqueous solution at a neutral pH of 7.4, with the hydrophilic macrocycles CD forming the surface of these NPs (Scheme 1A). Meanwhile, platelets (PLT) are modified with adamantane (Ada) by a straightforward membrane‐insertion method using 1,2‐distearoyl‐sn‐glycero‐3‐phosphoethanolamine‐N‐[methoxy (polyethylene glycol)‐2000]‐adamantane (DSPE‐PEG‐Ada).^[^ ^18^ ^]^ The CD‐functionalized NPs have the remarkable ability to securely anchor themselves to platelets via strong host‐guest interactions between multiple CD/Ada pairs.^[^ ^19^ ^]^ This interaction essentially acts as a lock mechanism, preventing any inadvertent release of the nanomedicine during circulation in the bloodstream and ensuring efficient targeted delivery to tumors through the recruitment of platelets caused by vascular injuries. Furthermore, when exposed to the acidic microenvironment of the tumor, these nanoformulations can transform into nanofibers (NFs), mimicking the role of fibrin in crosslinking platelets and neighboring red blood cells. This transformation facilitates the precise and effective occlusion of tumor blood vessels. In essence, our study offers a promising strategy for inducing tumor‐selective embolization by harnessing the conjugation of platelets and morphologically transformable nanostructures. This approach effectively mimics the natural thrombosis process by introducing artificial fibrin‐like structures within tumor vessels.

## 2 Results and Discussion

### 2.1 Synthesis and Characterization of Morphology‐Transformable Nanoparticles

**Figure 1:**
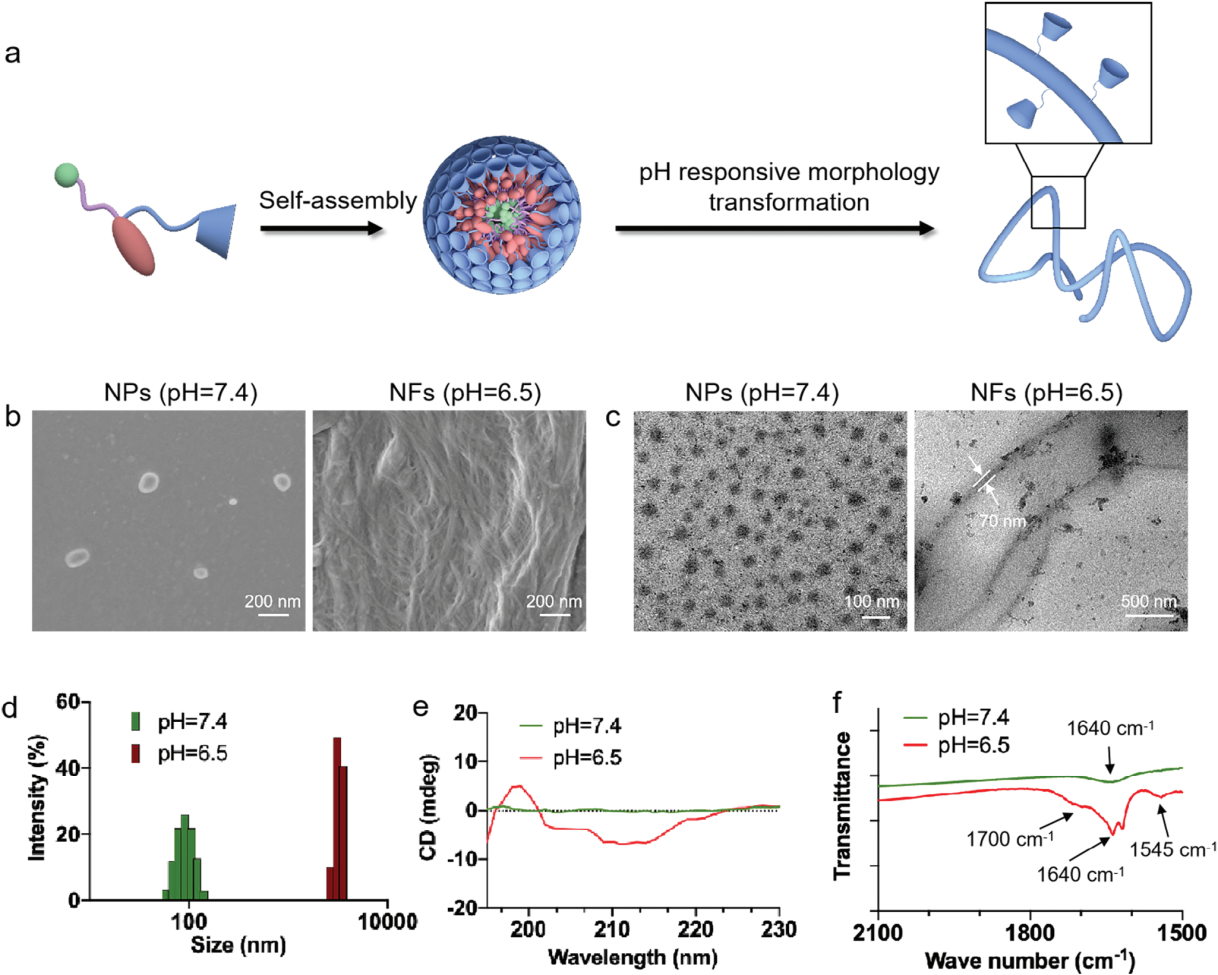
Synthesis and characterization of morphology‐transformable nanoparticles. a) Molecular structure of FHPC and schematic illustration of the morphological transformation of NPs into NFs. b) SEM and c) TEM images of NPs and NFs. Scale bar: 200 nm. d) DLS sizes, e) Circular dichroism spectra, and (f) Fourier transform infrared spectroscopy of NPs under different pH values.

To construct the pH‐responsive morphology‐transformable nanoplatforms, we first synthesized a hydrogen‐bonding peptide‐based system termed FHPC (**Figure** **1**a). The synthetic route of FHPC was depicted in Figure S1 (Supporting Information) and the successful synthesis was confirmed by ^1^H NMR (Figures S2–S4, Supporting Information). Specifically, in the realm of molecular design, a judiciously crafted motif was employed to confer the nanoparticles with the unique ability to steadily interact with platelets. The PEG chain, functionalized with CD, exhibited not only a propensity to interact favorably with surrounding water molecules, thereby establishing a stable interface in aqueous solution, but also served as a binding site to conjugate with platelets. Subsequently, FFVLK contributed to the vital morphology domination in deformable nanoparticles, due to its inherited fibril‐shaped self‐assembly via strong hydrogen bonding. Ultimately, benefiting hydrophobicity to hydrophilicity transition of the imidazole group in response to acidic conditions, His_6_ was introduced to endow the system with pH‐responsiveness. After all, the synthesized FHPC was dissolved in DMSO (20 mm), and adding into 99% water by rapid injection method as described in supporting information. The generated nanoparticles in an aqueous solution (pH = 7.4) exhibited a well‐defined spherical nanostructure as evidenced by SEM and TEM images (Figure 1–c, left). As designed, the protonation of His_6_ in acidic environment would heighten the hydrophilicity to disrupt the original amphiphilicity of nanoparticles. Thus, the dominant assembly force of FHPC would shift to FFVLK‐derived hydrogen bonding, leading to the morphology transformation from NPs to NFs. To validate this hypothesis, the amphiphilic NPs (20 µm) were added into PBS at pH 6.5. After 4 h incubation, a typical fibrous structure was observed by electron microscope images (Figure 1–c, right).

To corroborate the structural transformation, we employed dynamic light scattering (DLS) analysis. Size measurements revealed that NPs incubated within the normal physiological environment (pH = 7.4) exhibited monodispersity, displaying an average hydrodynamic diameter of ≈100 nm (Figure 1d). In contrast, when exposed to an acidic buffer (pH = 6.5), a notable change in size occurred, further validating the pH‐driven morphological transformation. The nanoparticles underwent a morphological transition into nanofibers under acidic conditions, while remaining stable as nanoparticles in neutral to alkaline environments (Figure S5, Supporting Information). The morphological transition of these peptide‐based nanomaterials is closely linked to alterations of secondary structures of peptides. A discernible negative peak ≈218–222 nm and a positive peak ≈195–200 nm was observed in circular dichroism (CD) spectra, as depicted in Figure 1e, demonstrating the β‐sheets secondary structure dominated by FFVLK after morphology transformation. Fourier transform infrared spectroscopy (FTIR) spectra further demonstrated that nanomaterials, when incubated in acidic environment, exhibited a complex structure of helix and antiparallel β‐sheets based on the amide I region at 1640 and 1700 cm^−1^ and amide II region peak at 1545 cm^−1^ (Figure 1f). All in all, these results strongly validated the hypotheses that FHPC molecules self‐assembled into nanoparticles possessing unique ability of undergoing in situ transformation into nanofibers in response to acid. Substantial evidence indicates that the perivascular regions of tumors exhibited slightly acidic pH conditions,^[^ ^5^, ^6^ ^]^ enabling this FHPC to selectively trigger embolization within the acidic tumor microenvironment.

### 2.2 Supramolecular Conjugation of Platelets and Nanoparticles

**Figure 2:**
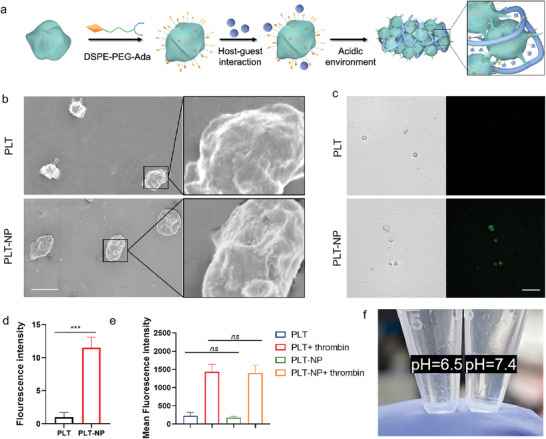
Supramolecular conjugation of platelet and nanoparticles (PLT‐NP). a) Preparation of PLT‐NP and schematic illustration of the coagulation process accelerated by acidity. b) SEM images of platelets with NPs anchored on membrane (untreated platelets served as control). Scale bar: 2 µm. c) CLSM images of PLT‐NP. Green channel for NPs. Scale bar: 20 µm. d) Quantitative analysis of the amount of NPs on platelets via flow cytometry (*n* = 3). NPs were labeled with Cy5.5. e) The expression of CD62P on platelets with different treatment (*n* = 3). f) Representative images of the clot formation of PLT‐NP treated by acidic buffer (pH = 6.5). Statistical significance was calculated by t‐test. *P* value: ^*^ *P* < 0.05, ^**^ *P* < 0.01, ^***^ *P* < 0.001.

We adopted a straightforward membrane‐insertion technique using DSPE‐PEG‐Ada to modify the platelet surface with Ada, offering platelet anchor sites to hold nanoparticles through the strong, multiple host‐guest interactions via CD/Ada pairs (**Figure** **2**a). Usually, platelets derived from Balb/c mice are sensitive to external stimuli, like temperature changes, mechanical vibrations, etc., and might be activated easily during extraction or modification. Accordingly, we found that the platelet activation degree was directly correlated with the concentration of DSPE‐PEG‐Ada in a dose‐dependent manner (Figure S6, Supporting Information). Thus, in addition to essential procedures to minimize platelet activation including anticoagulants appending, gentle handling, and prompt processing, we also carefully optimized the incubation concentration of DSPE‐PEG‐Ada, which was finally chosen as 1 µm to ensure an adequate amount of Ada modification and subsequent NPs conjugation, while also preventing undue platelet activation. The supramolecular formulations were generated by conjugating CD‐surfaced nanoparticles onto Ada‐functionalized platelets. The successful connection could be convincingly verified by the discernible presence of nanoparticles on the surface of platelets in comparison with the smooth appearance of natural platelets, demonstrated by SEM images in Figure 2b. To further confirm the conjugations, we prepared fluorescence‐labeled nanoparticles. As shown in the CLSM images (Figure 2c), platelets were surrounded by an obvious fluorescence signal, evidencing the successful surface modification of the particles. As indicated by the results of flow cytometric quantification analysis, the fluorescence intensity of Ada‐functionalized platelets exhibited a notable increase after mixing with nanoparticles, ≈11.5‐fold higher than that of natural platelets (Figure 2d; Figure S7, Supporting Information). To further quantify the conjugation efficiency, the fluorescence intensity of the nanoparticles was measured before and after conjugation to the platelets. After evaluating the reduction in fluorescence intensity, the conjugation ratio was calculated to be 25.96 ± 7.22% (Figure S8, Supporting Information). Moreover, the supramolecular conjugation stability was monitored for 24 h, using flow cytometry. The fluorescence intensity demonstrated a negligible decrease during this time frame, indicating that the PLT‐NP conjugates remained stable (Figure S9, Supporting Information). These findings provided compelling evidence of the substantial drug‐loading capacity of platelets after Ada‐ functionalization

We hypothesized that the PLT surface modified with NPs would preserve the typical physiological functions, thereby ensuring the PLT‐NP conjugates do not trigger undesired activation upon administration into the body, as well as initiating coagulation cascades when triggered by tumor‐specific embolization. By monitoring the expression of CD62P markers on platelets (Figure 2e; Figure S10, Supporting Information), the activation level of platelets conjugated with NPs did not exhibit significant differences from that of natural platelets, indicating the encapsulation of nanoparticles did not induce excessive platelet activation. Upon stimulation by thrombin, PLT‐NP demonstrated elevated expression levels of CD62P, similar to those of natural platelets. Moreover, the aggregation of untreated platelets and PLT‐NP under the stimulation of four agonists: arachidonic acid (AA), adenosine diphosphate (ADP), epinephrine (EPI), and collagen (COL) were evaluated. No significant difference was observed in aggregation rates between the two groups, suggesting that the nanoparticle‐loading did not adversely affect platelet aggregation capabilities (Figure S11, Supporting Information). These results indicated that the nanoparticles neither exert adverse effects on the physiological functionality of platelets nor impede the normal communication of platelets with surrounding signaling molecules, which offered a favorable indication regarding the in vivo active targeting potential of PLT‐NP, along with their ability to respond to thrombotic signaling and initiate sequential cascades of reactions. Subsequently, we exposed the PLT‐NP to an acidic buffer (pH = 6.5) to evaluate whether the pH reduction could induce embolization formation in vitro (Figure 2f). As anticipated, no noticeable clot formation was evident under phosphate buffer with pH 7.4, which implied that PLT‐NP eliminated the risk of nonselective thrombosis, consequently minimizing coagulation events and potential side effects in healthy tissues, which is a pivotal concern in tumor embolization therapy. In sharp contrast, prominent clot formation was observed when encountered acidic environment. These findings demonstrated the great potential of PLT‐NP to achieve selective embolization exclusively at tumor sites, while sidestepping vessel blockage in normal tissues, making PLT‐NP a high promising formulation for tumor suppression.

### 2.3 Active Recruitment of PLT‐ NP to Tumor Sites

**Figure 3:**
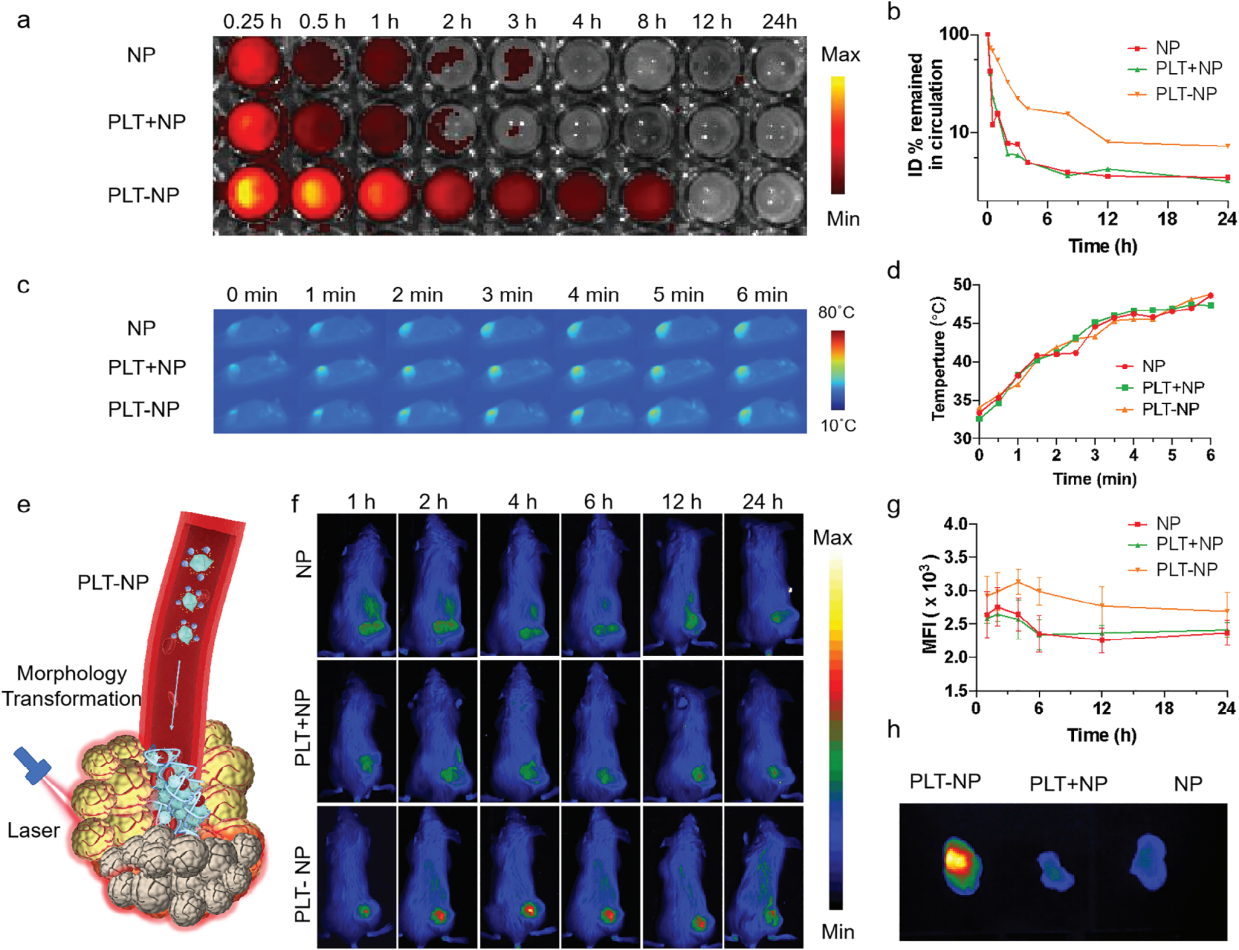
Active recruitment of PLT‐NP to tumor sites via vascular damage. a) Representative fluorescence images of the plasma obtained from healthy mice treated with NP, PLT+NP, and PLT‐NP, respectively. NPs were loaded with Cy5.5. b) Time course of NP levels in the blood of mice treated with NP, PLT+NP, and PLT‐NP, respectively. c) Representative thermal images of tumor‐bearing mice under irradiation and d) corresponding temperature changes. e) Schematic illustration of the targeting delivery and clot formation of PLT‐NP. f) In vivo fluorescence imaging and g) corresponding fluorescence intensities of tumor‐bearing mice post injection of NP, PLT+NP, and PLT‐NP, respectively. h) Ex vivo fluorescence imaging of tumors obtained from sacrificed mice 24 h post injection.

Attaching NPs to platelet surfaces has the potential to tune the in vivo pharmacokinetics of NPs. Hence, we measured the blood drug concentration at different time points in healthy mice administered with NP, the physical mixture of platelets and nanoparticles (PLT+NP), and the supramolecular conjugates of platelets and nanoparticles (PLT‐NP), respectively, using equivalent NPs dose. Fluorescence images of the blood obtained from mice at different time points revealed a significant extension in the circulation half‐life for NPs when tethered onto platelets (**Figure** **3**a), ≈7‐fold longer than that of control (Figure 3b), which mainly attributed to platelets' inherited immune evasion and prolonged circulation. The hyperthermia‐directed acute vascular damage was induced by a relatively low laser intensity (808 nm, 0.5 W cm^−2^), given that excessively high‐intensity irradiation may result in unwarranted damage to neighboring healthy tissues. According to the thermal imaging and quantitative temperature analysis of tumor sites, laser‐induced temperature raised properly to 45 °C (Figure 3c,d), a threshold previously reported to induce tumor vascular damage without inflicting excessive harm on the adjacent tissues.^[^ ^20^ ^]^

We subsequently evaluated their active targeting capability toward the tumor site, where vascular has been damaged by exposure to irradiation (Figure 3e). The tumor‐bearing mice were *i.v*. Injected with NP, PLT+NP, and PLT‐NP, respectively, with equivalent NPs dose. In vivo, fluorescence imaging was conducted at different time points post injection (Figure 3f), and the fluorescence intensity at tumor sites was quantitatively analyzed (Figure 3g). It was observed that nanoparticles exhibited enhanced accumulation in tumors when supramolecularly conjugated onto platelets, as evidenced by the significantly increased fluorescence signals in comparison with control group (NP and PLT+NP). Ex vivo fluorescence imaging of tumors and major organs further confirmed the maximum accumulation of PLT‐NP at the target sites (Figure 3h; Figure S12a, Supporting Information). Mice treated with PLT‐NP demonstrated 2.4‐fold higher fluorescence in tumors than those treated with the free NPs (Figure S12b, Supporting Information), which leveraged the inherent capability of platelets to home in on damaged blood vessels. To further confirm the high tumor accumulation was mainly attributed to platelet‐hitchhiking delivery, we conducted the fluorescence assays on frozen tumor sections to observe the localization of platelets and nanoparticles. Strong red fluorescence signals (NPs) were observed in tumors of mice injected with PLT‐NP, which exhibited a substantial overlay with the green channel (CD41 antibody used for platelet labeling). The overlapped florescence didn't tend to manifest as individual points but rather merged into extensive regions, implying that the PLT and NPs were strongly holding hand‐in‐hand together and formed clots when deposited in the tumors. Conversely, very modest red fluorescence was detected in tumors of mice treated with NP and PLT+NP, suggesting the restricted drug accumulation by passive targeting of nanoparticles (Figure S13, Supporting Information). Collectively, these findings demonstrated that PLT‐NP could efficiently target and accumulate at the tumor sites for subsequent clot formation, thus presenting substantial potential to augment vascular occlusion and enhance antitumor efficacy.

### 2.4 PLT‐NP Inhibited Tumor Growth Through In Situ Coagulation in Tumor Vessels

**Figure 4:**
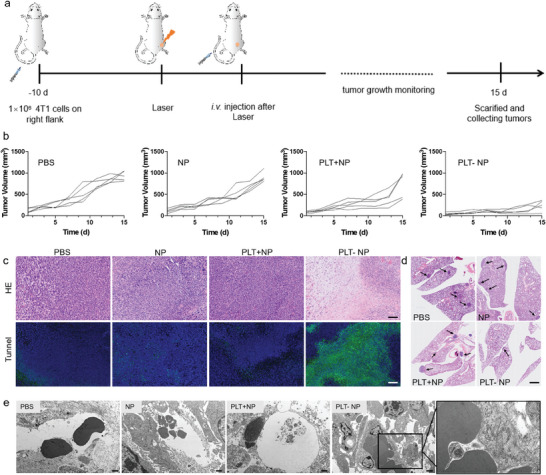
PLT‐NP inhibited tumor growth through in‐situ coagulation in tumor vessels. a) Schematic illustrating the PLT‐NP embolization therapy in tumor‐bearing mouse model. b) The growth curves of tumors in mice treated with PBS, NP, PLT+NP, and PLT‐NP, respectively (*n* = 5). c) H&E and tunnel staining of tumors. Scale bar: 200 µm. d) H&E staining of lungs from sacrificed mice with different treatments. Scale bar: 1 mm. The metastatic tumor cells were highlighted by arrows. e) Cryo‐TEM images of tumor sections, showing occlusion of tumor vessels (PLT‐NP). Scale bar: 200 µm.

The encouraging results mentioned above collectively support the notion that PLT‐NP demonstrated decent tumor targeting with prolonged circulation, with great potential serving as an effective tumor embolization therapeutic agent. In the laser‐induced vascular injury model, low‐power laser caused limited cell rupture and metabolic disruption, while most tumor cells remained in an acidic environment. This acidic condition facilitated the morphological transformation of the nanoparticles, leading to blockage of the tumor blood vessels. To verify the hypothesis, the anti‐tumor effect of PLT‐NP was further evaluated in vivo. Mice with damaged tumor vessels induced by irradiation were *i.v*. Injected with PBS, NP, PLT+NP, and PLT‐NP, respectively, as illustrated in **Figure** **4**a. Along with the notable capability to effectively target damaged tumors, the microenvironment responsiveness induced coagulation‐mimicking, specific and stable vascular embolization, finally leading to the significant suppressed tumor progression in PLT‐NP treated mice (Figure 4b; Figure S14, Supporting Information). The reduction of tumor weights observed in PLT‐NP group (Figure S15, Supporting Information), when compared to other groups, strongly implied that the selective occlusion of tumor blood vessels effectively hindered tumor growth. Moreover, histological examination of tumor tissues revealed evident physiological damages in the PLT‐NP treated group compared to the control group (Figure 4c). The condensed and fragmented nuclei observed in the TUNEL staining confirmed increased apoptosis in the embolization‐treated tumors, further supporting the anti‐tumor effect of PLT‐NP. Additionally, we also investigated the impact of embolization treatment on lung metastasis. H&E‐stained lung sections displayed a noticeable reduction in the number and size of lung metastatic lesions in the PLT‐NP group (Figure 4d), suggesting this therapeutic strategy could effectively suppress tumor metastasis to distant organs. Furthermore, the examination of tumor vascular occlusion through bio‐TEM analysis provided direct evidence of embolization‐induced blockade of tumor blood vessels (Figure 4e). Further verification through H&E sections of tumors also confirmed the in‐situ thrombus formation within tumor tissue as shown in Figure S16 (Supporting Information). Overall, our comprehensive analysis of anti‐tumor data collectively underscored the potential of PLT‐NP as a promising therapeutic strategy for tumor growth inhibition. The observed reductions in tumor size, increased apoptosis, suppression of lung metastasis, and successful tumor vascular occlusion validate the potential clinical significance of this approach in cancer treatment.

### 2.5 PLT‐NP Therapy Prevented Recurrence of Postsurgical Tumor

**Figure 5:**
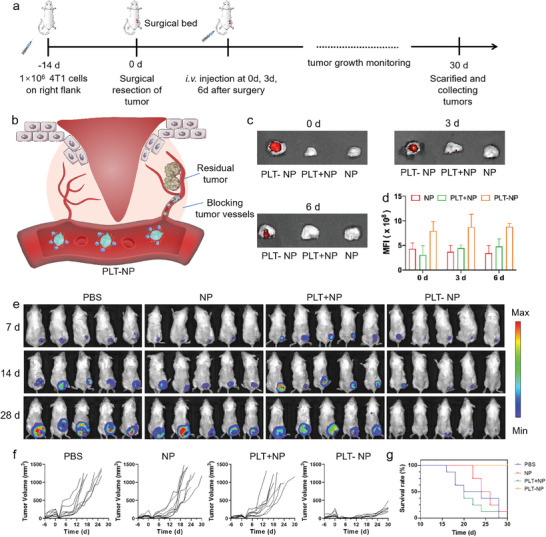
PLT‐NP therapy reduced tumor recurrence. a) Schematic illustration of the experiment protocol in incomplete‐surgery tumor model. b) Schematic illustration of the delivery of NPs to post‐surgical tumor sites by platelets. c) Ex vivo fluorescence images of tumor from mice injected with NP, PLT+NP, and PLT‐NP at 0 d, 3 d, and 6 d post‐surgery and d) corresponding quantitative analysis of fluorescence intensity. e) In vivo bioluminescence imaging of mice after removal of primary tumor. f) Tumor growth curves and g) survival rate of mice in different groups (*n* = 8).

Surgical resection remains one of the most frequently employed clinical treatments of tumors due to its potential for local tumor elimination and, in some cases, curative intent. However, despite the successful removal of the primary tumor, the risk of tumor recurrence persists as a significant challenge in cancer treatment. Few surgical techniques can guarantee complete eradication, as a result, tumor cells might tarry in nearby tissues or disseminate through the bloodstream or lymphatic system, giving rise to distant metastases.^[^ ^21^ ^]^ This phenomenon poses a substantial risk of disease relapse and underscores the need for complementary treatment strategies to address tumor recurrence.^[^ ^22^ ^]^ Neovascularization is closely linked to tumor recurrence due to its function of providing oxygen and nutrients, facilitating cancer metastasis and resistance.^[^ ^23^ ^]^ Thus, anti‐angiogenic therapies have been developed to disrupt the tumor's energy supply. Platelets can actively target to damaged blood vessels caused by surgical resection, making the platelet‐based conjugates enormous potential in inhibiting recurrence and metastasis of residual tumors. To validate the therapeutic efficacy of our strategy in post‐surgical tumors, the incomplete‐tumor‐resection model was employed to mimic post‐surgical local recurrence (**Figure** **5**a). As illustrated in Figure 5b, the artificial clots based on PLT‐NP conjugates were precisely constructed in situ in the residual tumor vasculature, which finally blocked the nutrient supply to the residual tumor and prevented the migration of tumor cells. To confirm the accumulation of NPs in residual tumors, NP, PLT+NP, and PLT‐NP were intravenously administered on mice at 0 days, 3 days, and 6 days post‐surgery (Figure 5c,d). The residual tumors were collected for fluorescence imaging 24 h after injection. Compared to tumors from mice injected with NP and PLT+NP, tumors from mice injected with PLT‐NP exhibited notably enhanced accumulation of nanoparticles, primarily attributed to the platelet's targeting ability toward injured blood vessels. In particular, after 24 h of injection, when the fluorescence of tumors in the other two groups was nearly undetectable, the PLT‐NP treated tumors still showed strong fluorescence, possibly due to the formation of artificial blood clots blocking blood vessels and preventing clearance of nanoparticles. The tumors of mice treated with PLT‐NP exhibited significant drug accumulation even when administered six days post‐surgery, indicating the exemplary tumor‐targeting proficiency.

The acidity of the tumor microenvironment is primarily due to the Warburg effect, where tumor cells rely on aerobic glycolysis, leading to excessive lactate production and accumulation.^[^ ^24^ ^]^ Although most of the tumor tissue was excised in post‐surgical tumor model, the remaining tumor cells typically continued to undergo high levels of glycolysis, producing lactate and causing local acidification. Additionally, the post‐surgical tissues remained hypoxic, which further encouraged the residual tumor cells to sustain or even exacerbate the acidic microenvironment. Based on the above results, we validated the effectiveness of this system in preventing tumor recurrence and metastasis in incomplete‐tumor‐resection mouse model. Balb/c mice were subcutaneously injected with 4T1 cells in the right rank. A major portion of the tumor was surgically resected after the tumor volume reached 100 mm^3^. Afterward, mice were randomly divided into four groups and intravenously administered with PBS, NP, PLT+NP, and PLT‐NP, respectively, at 0, 3, and 6 days post‐surgery. Tumor growth was monitored through the bioluminescence of 4T1 cells to track tumor progression with high precision and sensitivity. Remarkably, the data demonstrated a notable difference in bioluminescence intensity among the treatment groups (Figure 5e). The mice treated with PLT‐NP exhibited the lowest bioluminescence intensity, with 2 of 5 in this group showing undetectable tumors at 14 day. In sharp contrast, the mice treated with NP and PLT+NP showed strong bioluminescence signal, nearly comparable to PBS‐treated group, which suggested the treatment with nanoparticles alone or a physical mixture of nanoparticles and platelets, failed to suppress tumor recurrence. The tumor growth curves also confirmed the distinguished anti‐tumor properties of PLT‐NP (Figure 5f), which not only benefited from the targeted delivery of nanoparticles via platelets but also attributed to the in situ coagulation of the supramolecular conjugates precisely blocking tumor vessels. The survival time of PLT‐NP treated mice was significantly increased when compared with the control group (Figure 5g). Furthermore, PLT‐NP therapy substantially reduced lung metastasis, as confirmed by observation of the whole lungs and corresponding quantitative analysis of pulmonary metastatic nodules (Figure S17, Supporting Information). Meanwhile, the mice of all groups showed similar weight growth trends, indicating the favorable biocompatibility (Figure S18, Supporting Information). Moreover, to thoroughly assess the safety of platelet conjugates, the evaluation of in vivo toxicity was conducted on healthy mice. As shown in Figure S19 (Supporting Information), similar to the PBS group, there was no observable tissue damage in all main organs (Heart, Liver, Spleen, Lung, and Kidney) as indicated by H&E staining (Figure S19a, Supporting Information). Besides, no significant difference was observed in the liver and kidney function biomarkers between PLT‐NP‐treated mice and PBS‐treated mice. Additionally, hematological parameters, such as white blood cells (WBC), red blood cell count (RBC), lymphocytes (LYM), platelet count (PLT) et al., were all in the normal ranges (Figure S19b, Supporting Information). The coagulation function parameters, prothrombin time (PT), activated partial thromboplastin time (APTT), fibrinogen (FIB), and thrombin time (TT) were also evaluated (Figure S20, Supporting Information) to demonstrate that the injection of PLT‐NP did not induce obvious influence on these coagulation functions. These findings underscore the potential of our strategy in effectively addressing the challenges of tumor recurrence and metastasis following incomplete tumor resection.

## 3 Conclusion

In this study, the supramolecularly engineered platelets by conjugating β‐amyloid inspired morphology‐transformable nanoparticles onto platelets through supramolecular interactions were constructed, allowing for the on‐site creation of clots to specifically target and occlude tumor blood vessels. This pH‐responsive behavior was meticulously investigated in vitro and was subsequently explored for its anti‐tumor activities in vivo. We leveraged the natural recruitment of platelets during hemorrhage to actively concentrate the nanoparticles at the tumor site. Through a series of in vivo experiments using laser‐induced vascular injury and post‐surgical tumor‐bearing mouse models, we confirmed the precise accumulation of supramolecularly engineered platelets within the damaged tumor vessels and their effective vessel‐blocking abilities. In the laser‐induced vascular injury model, low‐power laser‐induced local vascular damage was utilized, followed by the targeted delivery of PLT‐NP, overcoming the limitations of current photothermal therapy, such as requirements of high energy laser that can damage the surrounding healthy tissues and the risk of tumor recurrence. The post‐surgical tumor model simulates the scenario of residual tumors following clinical surgery. By leveraging the natural targeting ability of platelets toward vascular damage, the targeting efficiency is enhanced, providing a new approach to addressing the challenge of tumor recurrence and metastasis after surgery. This system demonstrates significant potential, not only in effectively inhibiting tumor growth but also in preventing tumor recurrence after surgical removal, as well as in overcoming the significant challenge of unintentional embolization in non‐target tissues. By utilizing platelets, we gain dual advantages: the ability to transport nanoparticles to the target site for accumulation and the ability to actively participate in the coagulation process to locally block blood vessels. The resulting embolization not only restricts the supply of oxygen and nutrients to the tumor but also has the potential to hinder the migration of tumor cells through the vasculature, addressing critical aspects of tumor recurrence and metastasis.

Our study is primarily based on findings from a preclinical model, and it is imperative to emphasize that further investigations are required to fully assess the translational potential of this strategy in clinical settings. These steps include rigorous safety assessments, optimization of treatment protocols, and a thorough examination of potential adverse effects. Additionally, it would be interesting to explore potential interactions of this approach with existing therapeutic modalities to determine the possibility of synergistic effects. The combined advantages of specific accumulation at the tumor site and effective vessel occlusion provide a compelling foundation for further exploration and development. This may lead to the emergence of innovative approaches in cancer therapy.

## 4 Experimental Section

### Materials

Hexahistidine and Fmoc‐FFVLK was purchased from Bankpeptide Biological Technology Co., LTD. Hydrochloride (EDC) and N‐hydroxysuccinimide (NHS) were sourced from Sigma–Aldrich (China). NHS‐PEG (2000)‐NHS was purchased from Xi'an Ruixi Biological Technology Co., LTD. All other reagents and solvents employed in this study were commercially available and utilized without further purification. Milli‐Q water was purified using a Milli‐Q Integral system from Merck Millipore. ^1^H NMR spectrum was acquired on a Bruker Ultra Shield 600 PLUS NMR spectrometer. The DLS sizes were determined with a Zeta Sizer (Malvern. Co., UK). SEM analysis was performed by a Zeiss Sigma. TEM analysis was performed by a transmission electron microscopy (TEM, JEOL 2100F, Japan) at the operation voltage of 200 kV. Visualization of the supramolecular conjugates was performed via Confocal Laser Scanning Microscopy (CLSM, Leica TCS SP8).

### Cell Lines and Animals

The luciferase‐tagged 4T1 and 4T1 cell lines were purchased from the American Type Culture Collection (ATCC, Shanghai, China). All animal procedures were performed following the Guidelines for Care and Use of Laboratory Animals of the University of Macau (protocol ID: APP‐ARE‐045).

### Synthesis of FHPC and Preparation of Nanoparticles

The NHS‐PEG‐NHS (40 mg, 20 µmol) was dissolved in 2 mL DMSO and then added, dropwise, NH_2_‐CD (11.34 mg, 10 µmol) allowing the reaction to react for 24 h then compound PC was obtained after 24 h of dialysis. The peptide sequence, FFVLK (10.5 mg; 12 µmol) was added to PEG‐CD chain (31.34 mg, 10 µmol) and the mixture was stirred for 24 h to obtain FPC. The product was obtained through connecting hexahittidine (10 mg; 12 µmol) with FPC (40 mg; 10 µmol). The final product FHPC was obtained after dialysis and lyophilization.

The obtained FHPC was dissolved in DMSO (20 mm) and added into 99% water by rapid injection method. The prepared nanoparticles were used for DLS size analysis and morphology observation. The modulation with acidic buffer (6.5) for hours triggered the formation of nanofibers. The morphology transformation of nanoparticles to nanofibers was confirmed via size measurement, CD spectrum, FTIR spectrum, and morphology observation via SEM/TEM.

### Preparation of Supramolecular Conjugations

The platelets were extracted from mice as previously reported. First, whole blood was collected from anesthetized Balb/c mice into a 1.5 mL EP tube containing anticoagulant solution. Then platelet‐rich plasma (PRP) was collected from the supernatant after centrifugation of whole blood at 100 g for 20 min. PGE1 at 1 µm was added to inhibit platelet activation. To isolate platelet precipitates, the PRP was centrifugated at 800 g for 15 min. The platelets were functionalized with Ada via membrane‐insertion with DSPE‐PEG‐Ada (1 µm, 1.5 h at 37 °C). After centrifugation to remove excess DSPE‐PEG‐Ada, platelets (1 × 10^7^) were dispersed in 500 µl of PBS including PGE1, and mixed with prepared nanoparticles. After 5 min mixture, the excessive nanoparticles were removed.

### Verification of the PLT‐NP via SEM, CLSM and Flow Cytometry

Before observing cells using SEM, a series of steps are undertaken to prepare the cells for imaging. Initially, cells are fixed using a suitable fixative, 2.5% glutaraldehyde, to preserve their morphology. Subsequently, dehydration was carried out through a graded ethanol series. After critical point drying or using a CO_2_ process, the dehydrated cells were coated with a thin layer of conductive material like gold or platinum. This ensures proper electron conductivity during SEM imaging, enabling detailed and high‐resolution visualization of cell surfaces and structures.

The NPs were fluorescently labeled for CLSM observation and flow cytometric analysis. After mixing platelets with nanoparticles and washing away excess nanoparticles, observation can be carried out using fluorescence microscopy. The presence of nanoparticles on the platelet surface can be confirmed through surrounding fluorescence, indicating successful binding. Following PLT‐NP complex formation, a semi‐quantitative analysis of particle density was performed using flow cytometry.

### Analysis of the Conjugation Ratio

The fluorescence intensity of the Cy5.5‐labeled nanoparticles was measured using a Fluorescence Spectrophotometer. This provided a baseline measurement for the total amount of labeled nanoparticles present before conjugation. Subsequently, the Cy5.5‐labeled nanoparticles were incubated with the platelets to allow for conjugation. After the conjugation process, the mixture was centrifuged to separate the platelet conjugates from unbound nanoparticles in the supernatant. The fluorescence intensity of the supernatant was measured to determine the amount of unbound nanoparticles remaining. Finally, the reduction in absorbance before and after conjugation was used to calculate the conjugation ratio.

### Analysis of Platelet Activation

Platelets (PLT) or platelets conjugated nanoparticles (PLT‐NP) were incubated with CD62 antibodies for surface binding (1 h, 37 °C), potentially using fluorescent labeling, and then subjecting the samples to flow cytometric analysis. The flow cytometer excites the labeled platelets, measuring their fluorescence and scattering signals to determine activation status.

To activate the platelets, thrombin (0.5 U mL^−1^) was added into the suspension of PLT or PLT‐NP (30 min, 37 °C). Afterward, platelets were incubated with CD62P antibodies and detected using flow cytometry.

### Platelet Aggregation Assay

The whole blood was collected into anticoagulant‐treated tubes to prevent clotting. The PRP was obtained from whole blood through centrifuging at a low‐speed spin (100 g, 20 min), and platelet‐poor plasma (PPP) was obtained by a high‐speed spin (800 g, 15 min). 200 µL of PRP was added to a 96 well plate, and 10 µL of different agonists, ADP (10 µm), COL (30 µg mL^−1^), AA (1 mm), and EPI (20 µm). were added to the PRP to induce aggregation. The absorbance of PPP solution and the PRP solution before and after the addition of agonist at 570 nm was measured by a microplate reader. Platelet aggregation rate was calculated as follow: [1‐(PRP absorbance value after addition of agonist‐ PPP absorbance value/(PRP absorbance value before the addition of activator‐ PPP absorbance value)] × 100%. To evaluate the effect of nanoparticle loading on platelet aggregation behavior, PRP was pre‐incubated with DSPE‐PEG‐Ada and NPs before the addition of agonists.

### In Vitro Evaluation of Clot Formation

The prepared PLT‐NP was added in 1.5 mL EP tubes containing acidic buffer (pH = 6.5) and normal buffer (pH = 7.4), respectively. After 4 h incubation, the state of PLT‐NP in different buffer solutions was observed.

### In Vivo Pharmacokinetics

Balb/c mice aging 6 weeks were divided into 3 groups (n = 3) and *i.v*. Injected with100 µL of NP, physical mixture of platelets and nanoparticles (PLT+NP) and supramolecular conjugates of platelets and nanoparticles (PLT‐NP), respectively (the number of platelets is 1 × 10^7^; NPs were labeled with Cy5.5). 100 µL of blood were collected from mice at 0.25, 0.5, 1, 2, 3, 4, 8, 12 and 24 h post injection. Ex vivo fluorescence images of the blood samples were obtained using the IVIS Spectrum system.

### Tumor Models and Treatments

Balb/c mice aging 6 weeks were subcutaneously injected with 4T1 tumor cells in the right rank (1 × 10^6^ cells; 100 µL). When the tumor volume reached 100 mm^3^, the mice were randomly divided into four groups (*n* = 6): PBS, NP, PLT+NP, and PLT‐NP. Injections were administered every three days for a total of three injections. Prior to each injection, the tumor site was exposed to laser irradiation (808 nm, 0.5 W cm^−2^) to induce vascular damages. The tumor volumes were measured with a digital caliper (tumor volume: long diameter × short diameter^2^/2).

### Fluorescence Imaging and Immunofluorescence Analysis

Balb/c mice aging 6 weeks were subcutaneously injected with 4T1 tumor cells in the right rank (1 × 10^6^ cells; 100 µL). When the tumor volume reached 100 mm^3^, the mice were randomly divided into 3 groups (*n* = 3): NP, PLT+NP, and PLT‐NP. (NPs were labeled with Cy5.5 to track their in vivo biodistribution). At various time points post‐injection, the mice were anesthetized, and fluorescence images of the tumor sites were captured. After 24 h, the mice were euthanized, and tumors and major organs were collected and imaged. The fluorescence intensities of the tumors and organs were quantitatively analyzed.

### Incomplete‐Tumor‐Resection Model

Balb/c mice aging 6 weeks were subcutaneously injected with 4T1 tumor cells in the right rank (1 × 10^6^ cells; 100 µL). When the tumor volume reached 100 mm^3^, the mice were anesthetized using isoflurane. Then tumors were resected, leaving ≈1% residuals in the surgical bed. After all, sutures were employed to hold wounds closed. These mice were randomly divided into 4 groups immediately after surgery (eight mice for each group) and intravenously injected with one of the following formulations: PBS, NP (0.02 µmol), PLT+NP (0.02 µmol NP psychically mixed with 1 × 10^7^ platelets), and PLT‐NP (0.02 µmol NP conjugated on 1 × 10^7^ platelets). Injections were administered every three days for a total of three injections. The tumor volumes were measured over a month.

To visualize the metastatic lung tumor, freshly collected lungs from sacrificed mice were intratracheal injected with India ink. Gently manipulate the lungs to ensure that the ink solution is evenly distributed throughout the lung tissue which helps visualize the lung structure and metastatic nodules. Several hours post fixation, the normal tissues were stained to black while tumors remain bleached.

The progression of tumor burden was tracked by monitoring the bioluminescence emitted by the cancer cells. Balb/c mice aging 6 weeks were subcutaneously injected with luciferase‐tagged 4T1 tumor cells in the right rank (1 × 10^6^ cells; 100 µL). During the treatment, mice were employed for bioluminescence imaging. The progression of tumor burden can be tracked via analysis of the captured images and quantification of the bioluminescent signal.

### Statistical Analysis

All data are expressed as the mean ± SD. Biological replicates were used in all experiments. For statistical analysis of the samples were performed using unpaired two‐tailed Student's t‐test and *P* value of <0.05 was considered significant.

## Conflict of Interest

The authors declare no conflict of interest.

## Supporting information



Supporting Information

## Data Availability

The data that support the findings of this study are available from the corresponding author upon reasonable request.
