# Supplementary material for: Tumor Microenvironment Triggered In Situ Coagulation of Supramolecularly Engineered Platelets for Precise Tumor Embolization
Source: Adv Sci (Weinh). 2025 Apr 7;12(26):2414879. doi: 10.1002/advs.202414879 (PMC12245090; doi:10.1002/advs.202414879)
Supplement: Supplementary file 1 — Supporting Information [file ADVS-12-2414879-s001.docx]

Supporting Information

**Tumor microenvironment triggered in-situ coagulation of supramolecularly engineered platelets for precise tumor embolization**

Junyan Li^a^, Ziyi Wang^a^, Ruifeng Luo^a^, Xingping Quan^a^, Hong U Fong^a^, Qian Cheng^a^, Jianwen Wei^a^, Leo Wang^a,c^, Yonghua Zhao^a^, Ruibing Wang^a,b,*^

**
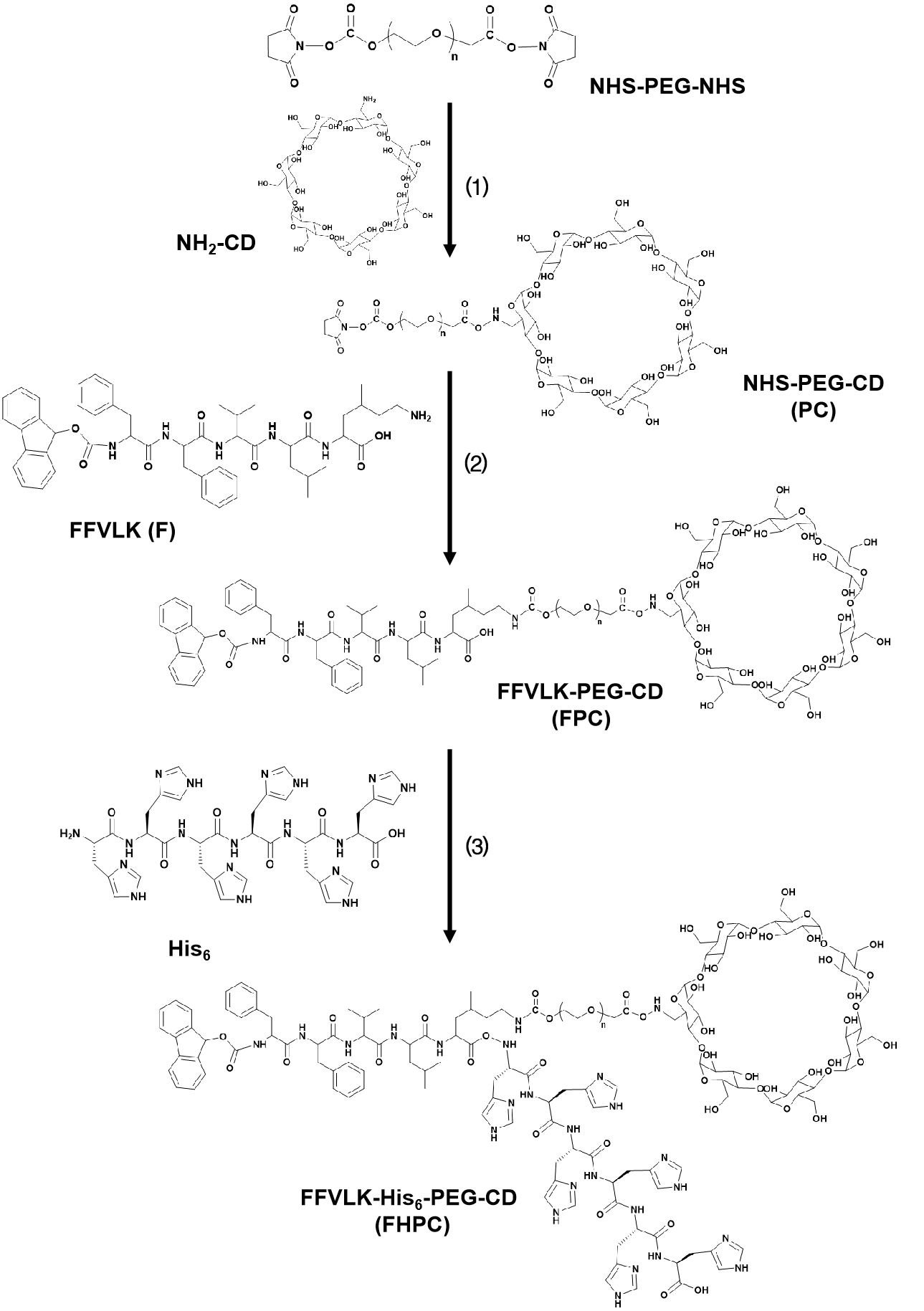
**

**Figure S1**. Schematic representation of HFPC.


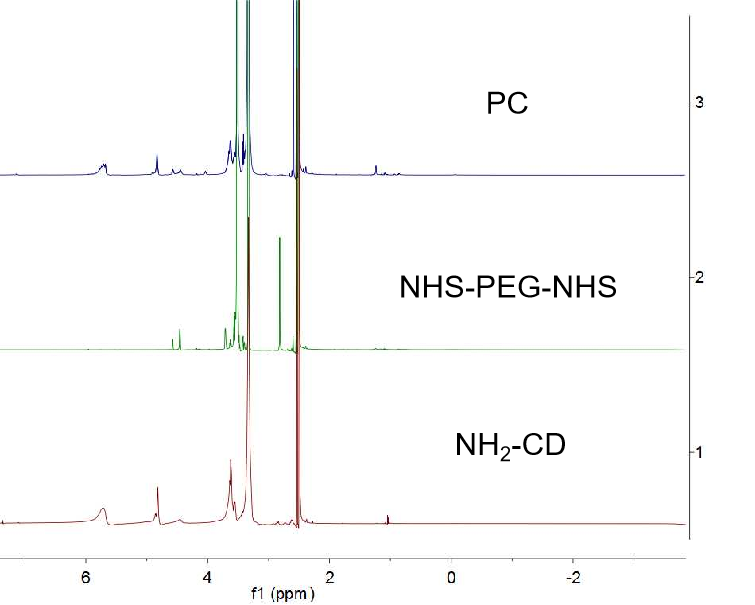


Figure S2. ^1^H NMR spectra of NH_2_-CD, NHS-PEG-NHS and NHS-PEG-CD (PC).


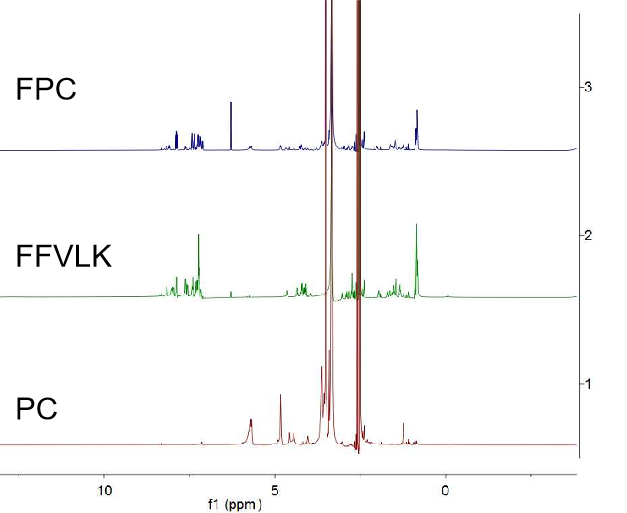


Figure S3. ^1^H NMR spectra of NHS-PEG-CD (PC), FFVLK and FFVLK-PEG-CD (FPC).


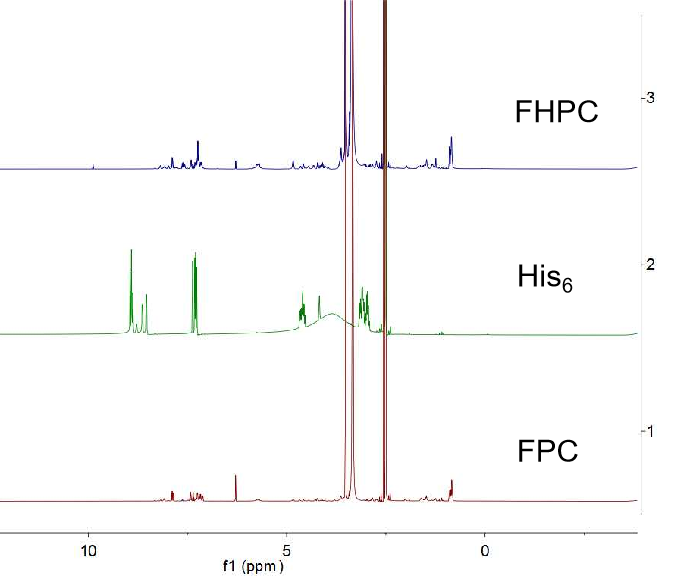


Figure S4. ^1^H NMR spectra of FFVLK-PEG-CD (FPC), His_6_ and FFVLK-His_6_-PEG-CD (FHPC).


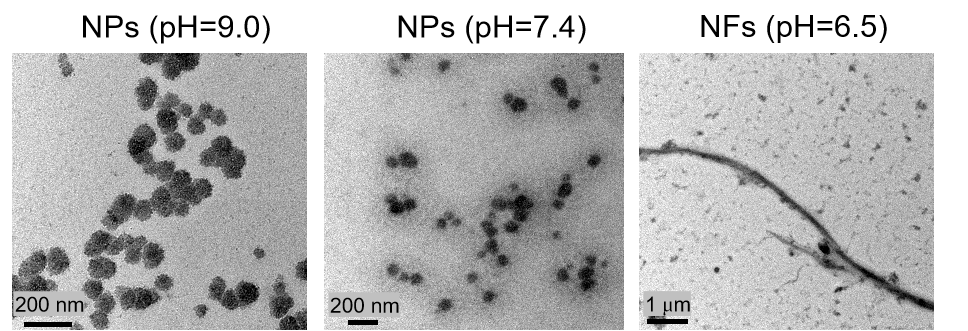


**Figure S5.** The TEM images of nanomaterials under different pH.


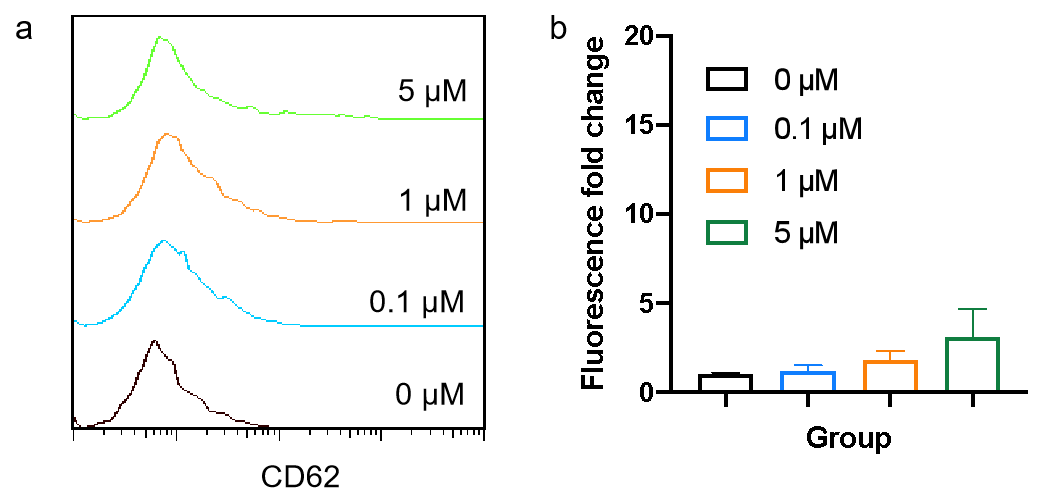


Figure S6. (a). Detection of the expression of CD62P on platelets incubated with DSPE-PEG-Ada at different concentration, via flow cytometry and (b) corresponding quantitative analysis of fluorescence intensity. Data are expressed as the mean ± SD.


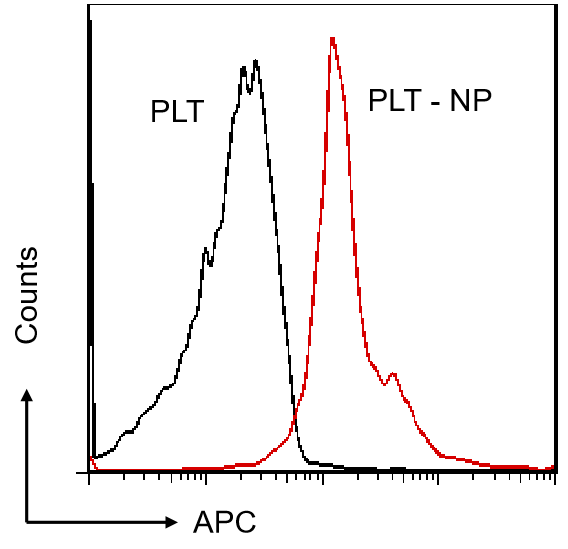


Figure S7. Detection of the amount of NPs anchored on platelets via flow cytometry.


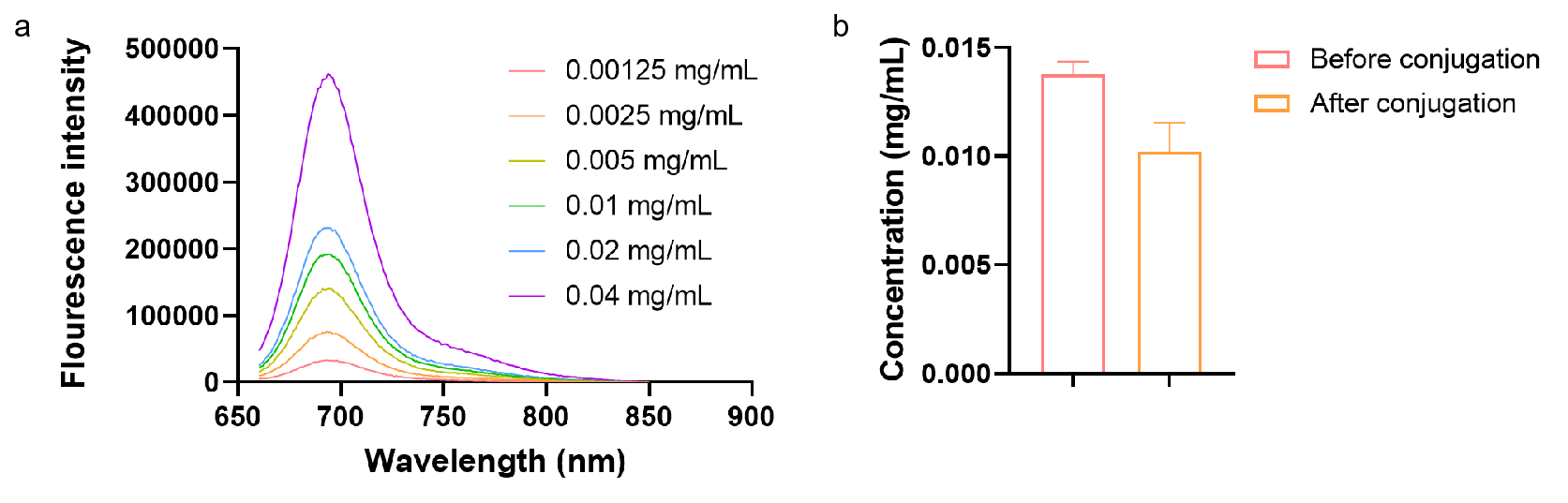


**Figure S8.** (a) Detection of the fluorescence intensity of Cy5.5 at different concentration and (b) the corresponding concentration of Cy5.5 among Cy5.5-labeled nanoparticles before and after conjugation. Data are expressed as the mean ± SD.


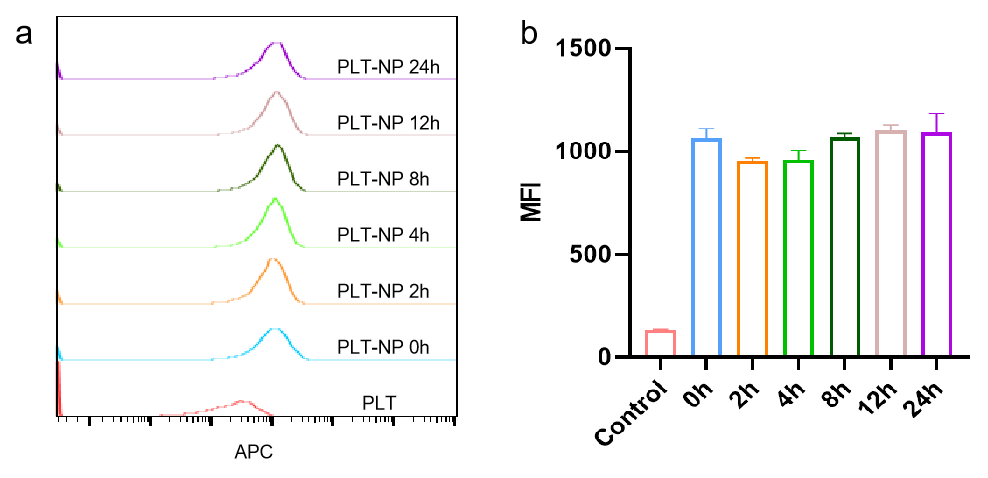


**Figure S9**. (a). Detection of the fluorescence intensity of platelet-nanoparticle conjugates at different time points by flow cytometry and (b) corresponding quantitative analysis of fluorescence intensity. Data are expressed as the mean ± SD.


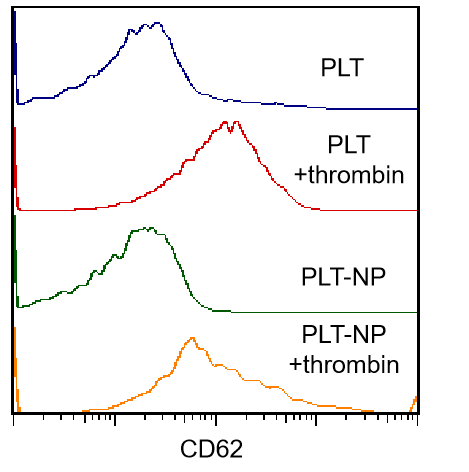


Figure S10. Detection of the expression of CD62P on platelets with different treatments, via flow cytometry.


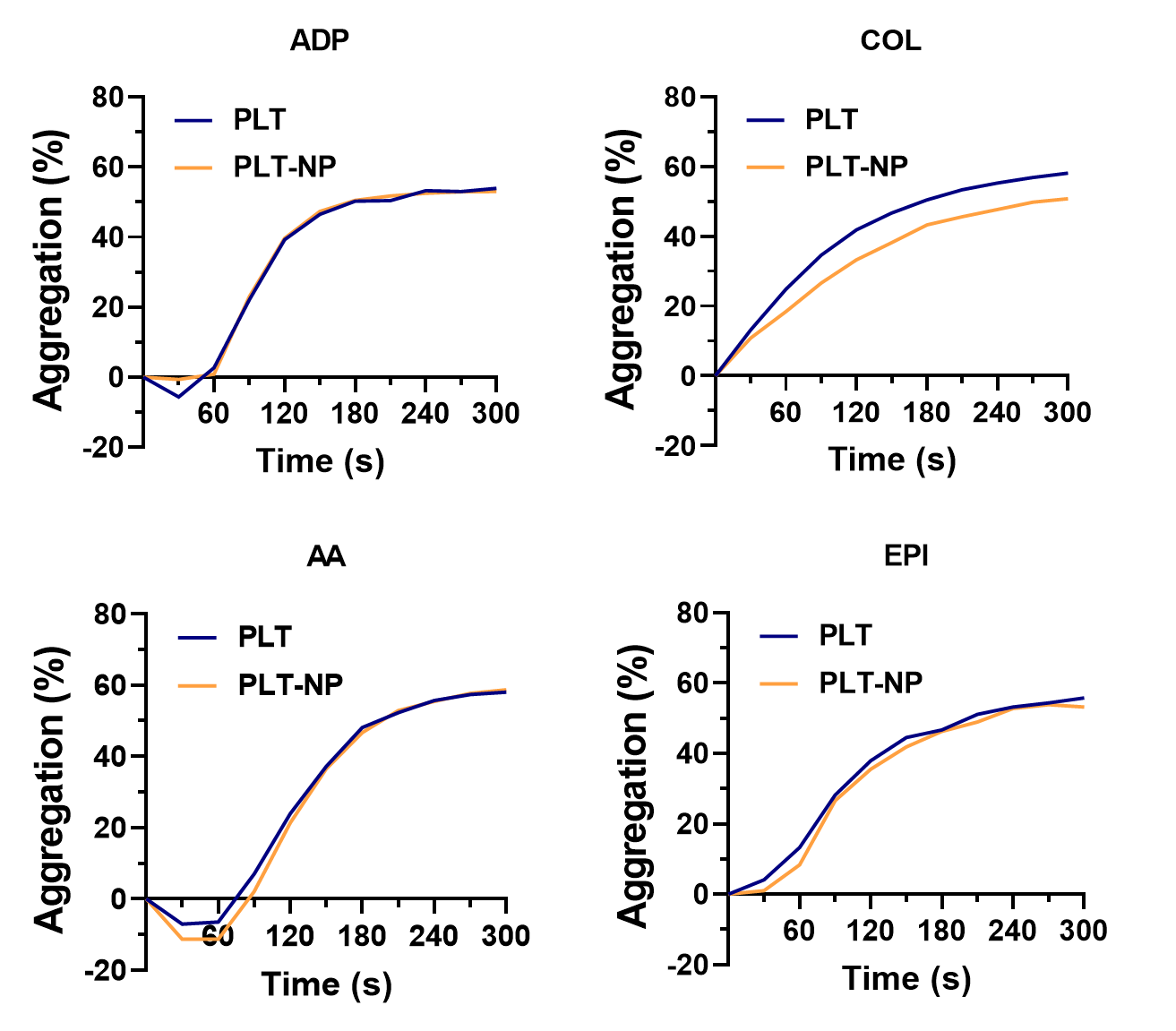


Figure S11. The platelet aggregation induced by ADP (10 μM), COL (30 μg/mL), AA (1 mM), and EPI (20 μM).


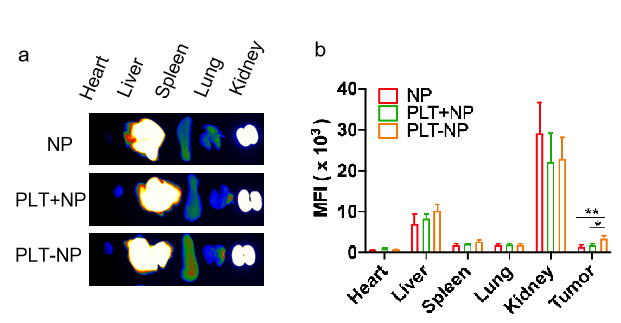


Figure S12. (a).Ex vivo fluorescence imaging of tumors obtained from sacrificed mice 24 h post injection. (b) Quantitative analysis of fluorescence intensity of organs and tumors (n=3).Data are expressed as the mean ± SD. Statistical significance was calculated by t-test. P value: *P<0.05, **P<0.01, ***P<0.001.


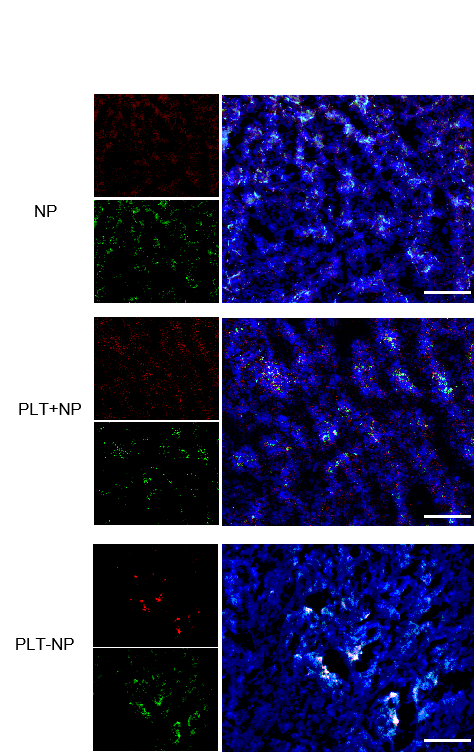


Figure S13. Frozen sections of tumor from mice 24 h post injection of NP, PLT+NPs and PLT-NPs. Red, green and blue channels represent NPs, platelets and nucleus, respectively. Scale bar: 100 μm.


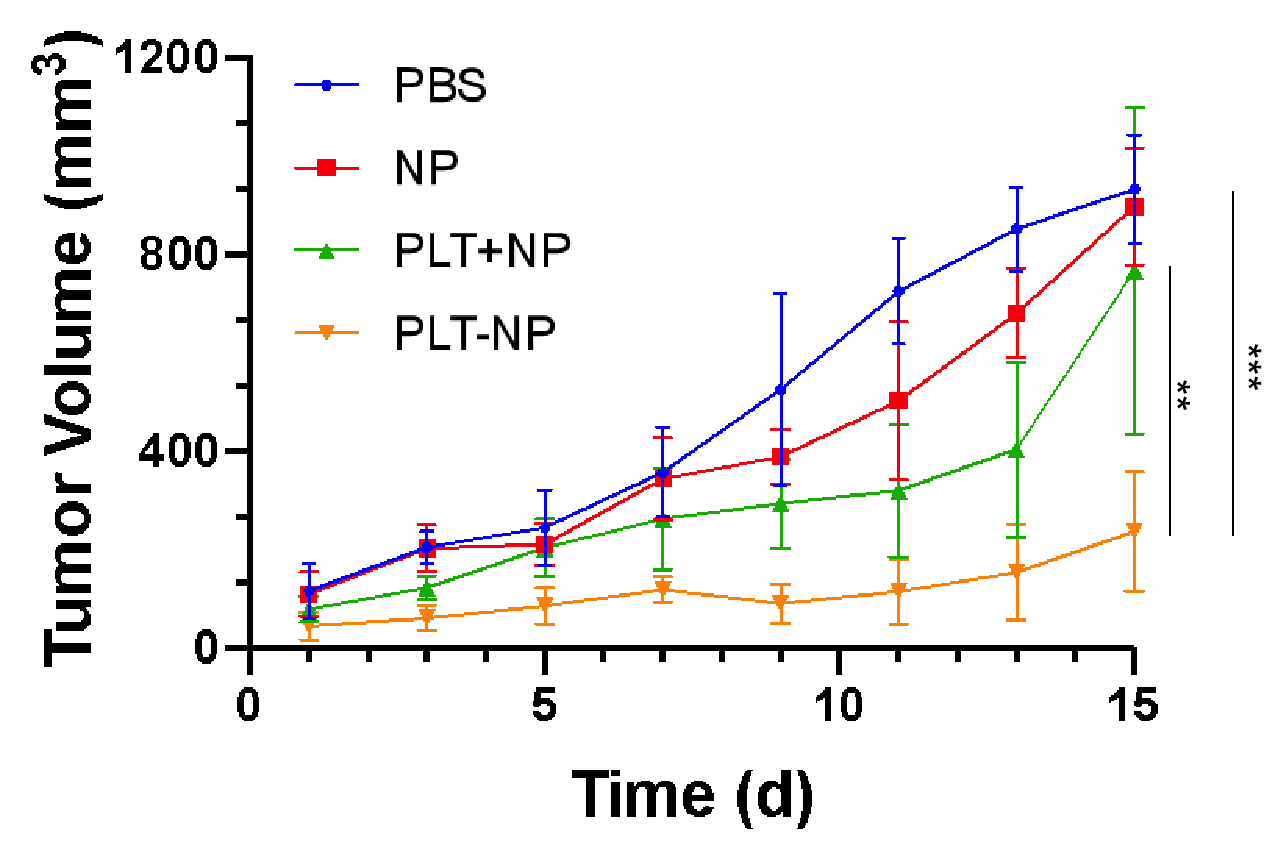


Figure S14. The growth curves and weight of tumors in different groups (n=5). Data are expressed as the mean ± SD. Statistical significance was calculated by t-test. P value: *P<0.05, **P<0.01, ***P<0.001.


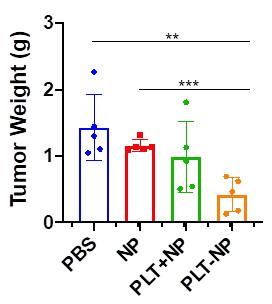


Figure S15. The weight of tumors in different groups (n=5). Data are expressed as the mean ± SD. Statistical significance was calculated by t-test. P value: *P<0.05, **P<0.01, ***P<0.001.


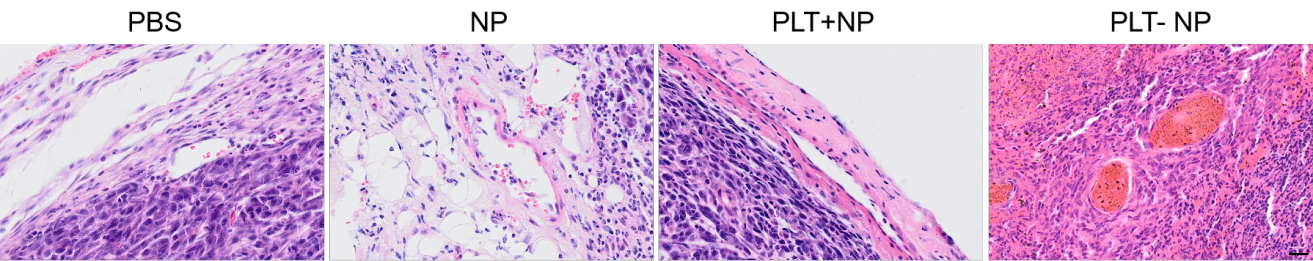


**Figure S16**. Hematoxylin and Eosin (H&E) staining of tumor vasculature in different groups.Scale bar: 20 μm.


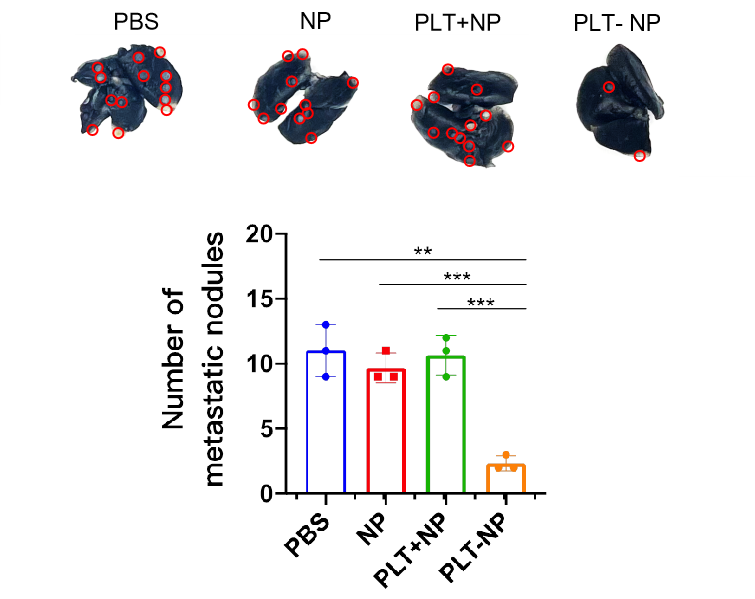


Figure S17. The lung images showing tumor metastasis of mice and corresponding quantitative analysis (n=3). Data are expressed as the mean ± SD. Statistical significance was calculated by t-test. P value: *P<0.05, **P<0.01, ***P<0.001.

#
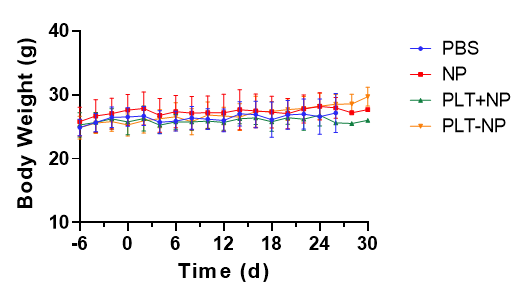


Figure S18. The body weight changes of mice in different groups.


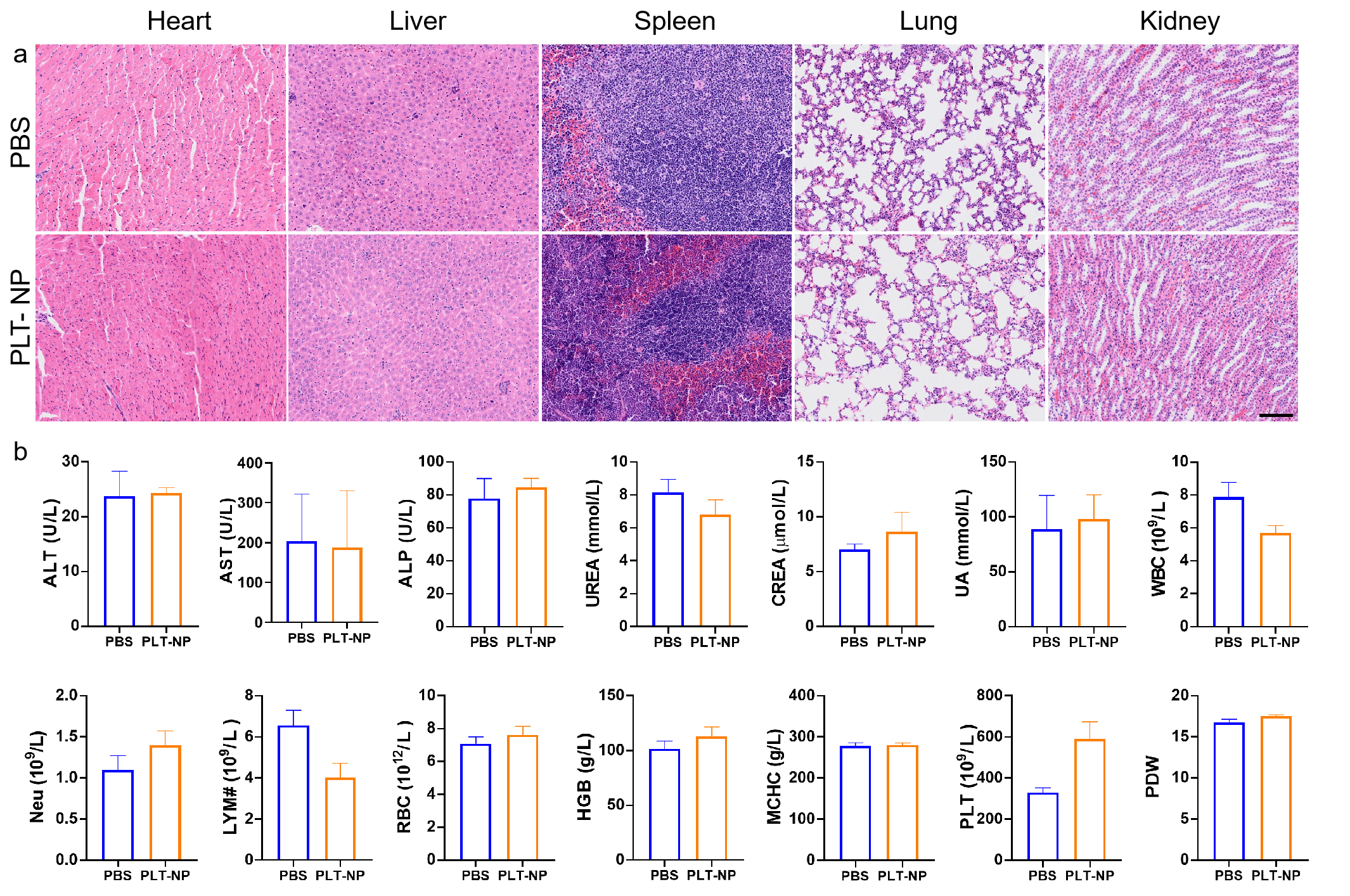


Figure S19. Assessment of *in vivo* toxicity. a) H&E staining of main organs and b) blood biochemical levels and hematological parameters of mice intravenously injected with different formulations 24 hours post-injection. Scale bar: 100 μm.


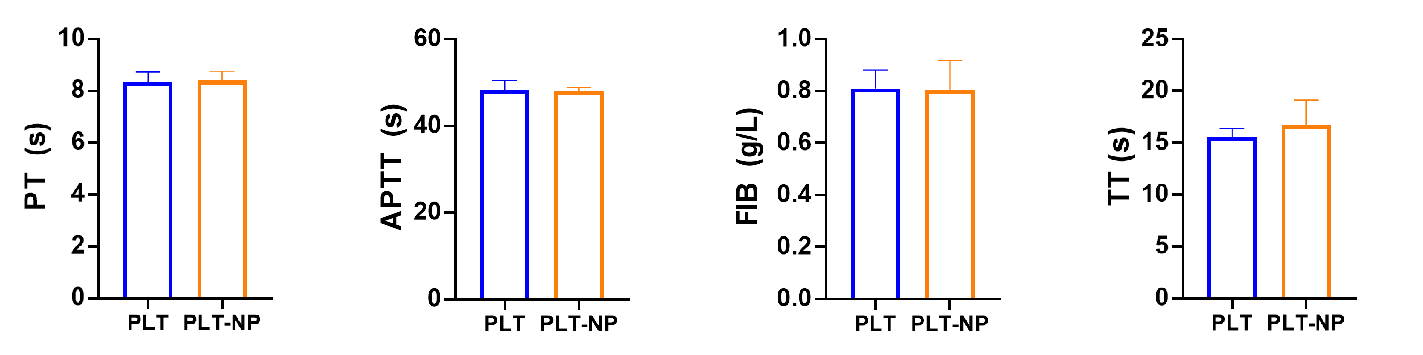


**Figure S20.** Assessment of the coagulation function. Prothrombin time (PT), activated partial thromboplastin time (APTT), the level of fibrinogen (FIB) and thrombin time (TT).
